# Identification and *In Vivo* Characterization of NvFP-7R, a Developmentally Regulated Red Fluorescent Protein of *Nematostella vectensis*

**DOI:** 10.1371/journal.pone.0011807

**Published:** 2010-07-27

**Authors:** Aissam Ikmi, Matthew C. Gibson

**Affiliations:** 1 Stowers Institute for Medical Research, Kansas City, Missouri, United States of America; 2 Department of Anatomy and Cell Biology, Kansas University Medical School, Kansas City, Kansas, United States of America; Katholieke Universiteit Leuven, Belgium

## Abstract

**Background:**

In recent years, the sea anemone *Nematostella vectensis* has emerged as a critical model organism for comparative genomics and developmental biology. Although *Nematostella* is a member of the anthozoan cnidarians (known for producing an abundance of diverse fluorescent proteins (FPs)), endogenous patterns of *Nematostella* fluorescence have not been described and putative FPs encoded by the genome have not been characterized.

**Methodology/Principal Findings:**

We described the spatiotemporal expression of endogenous red fluorescence during *Nematostella* development. Spatially, there are two patterns of red fluorescence, both restricted to the oral endoderm in developing polyps. One pattern is found in long fluorescent domains associated with the eight mesenteries and the other is found in short fluorescent domains situated between tentacles. Temporally, the long domains appear simultaneously at the 12-tentacle stage. In contrast, the short domains arise progressively between the 12- and 16-tentacle stage. To determine the source of the red fluorescence, we used bioinformatic approaches to identify all possible putative *Nematostella* FPs and a *Drosophila* S2 cell culture assay to validate NvFP-7R, a novel red fluorescent protein. We report that both the mRNA expression pattern and spectral signature of purified NvFP-7R closely match that of the endogenous red fluorescence. Strikingly, the red fluorescent pattern of NvFP-7R exhibits asymmetric expression along the directive axis, indicating that the *nvfp-7r* locus senses the positional information of the body plan. At the tissue level, NvFP-7R exhibits an unexpected subcellular localization and a complex complementary expression pattern in apposed epithelia sheets comprising each endodermal mesentery.

**Conclusions/Significance:**

These experiments not only identify NvFP-7R as a novel red fluorescent protein that could be employed as a research tool; they also uncover an unexpected spatio-temporal complexity of gene expression in an adult cnidarian. Perhaps most importantly, our results define *Nematostella* as a new model organism for understanding the biological function of fluorescent proteins *in vivo*.

## Introduction

Fluorescent proteins derived from cnidarians have revolutionized biomedical research by potentiating the detailed analysis of protein localization and dynamics in real time [1]. However, we still know relatively little about endogenous expression and function of these molecules *in vivo*. The sea anemone *Nematostella vectensis* is an emerging cnidarian model system, which is primarily used to provide insight into the evolution of genetic and morphological complexities in animals [2], [3], [4], [5], [6]. *Nematostella* embryos can be obtained in vast quantities, and develop rapidly into swimming planula larvae that undergo a transition into juvenile polyps within ten days of development. The polyps then progressively add tentacles in concert with growth, reaching 12–16 tentacles at sexual maturity. Besides the fact that it can be easily maintained and spawned in laboratory conditions [7], [8], *Nematostella* has an ideal phylogenetic position within cnidarians, a sister group to the Bilateria. The *Nematostella* genome also exhibits a low degree of sequence evolution, as evidenced by a high level of conservation of ancestral genetic traits that are likely inherited from the last common ancestor with Bilateria [5], [9]. Based on these and other considerations, *Nematostella* is considered an attractive model to capture the ancient functions of conserved genes and signalling pathways among the Metazoa.

To develop a mechanistic understanding of *Nematostella* development, several functional approaches have been established including morpholino-based gene knockdowns and gene overexpression [2], [10], [11]. Recently, transgenic *Nematostella* expressing the red fluorescent protein mCherry have been generated [12], opening the possibility of using live imaging to monitor the cellular and developmental processes driving *Nematostella* growth and morphogenesis. Given the fact that GFP-like proteins are abundant and diverse within Cnidaria [13], [14], [15], a critical unanswered question is whether the *Nematostella* genome contains any member of the GFP family that should be considered in light of experiments utilizing induced fluorescence. In fact, multiple fluorescent proteins have been isolated from a single cnidarian species [16], [17], [18], raising the following questions: How many and what kind of GFP-like genes are encoded by the *Nematostella* genome? When and where they are expressed? And finally, what developmental or environmental factors could trigger the expression of these genes? Here, we explore these issues through characterization of a novel red fluorescent protein isolated from *Nematostella*.

## Results

### Patterned expression of endogenous fluorescence in adult *Nematostella*

**Figure 1:**
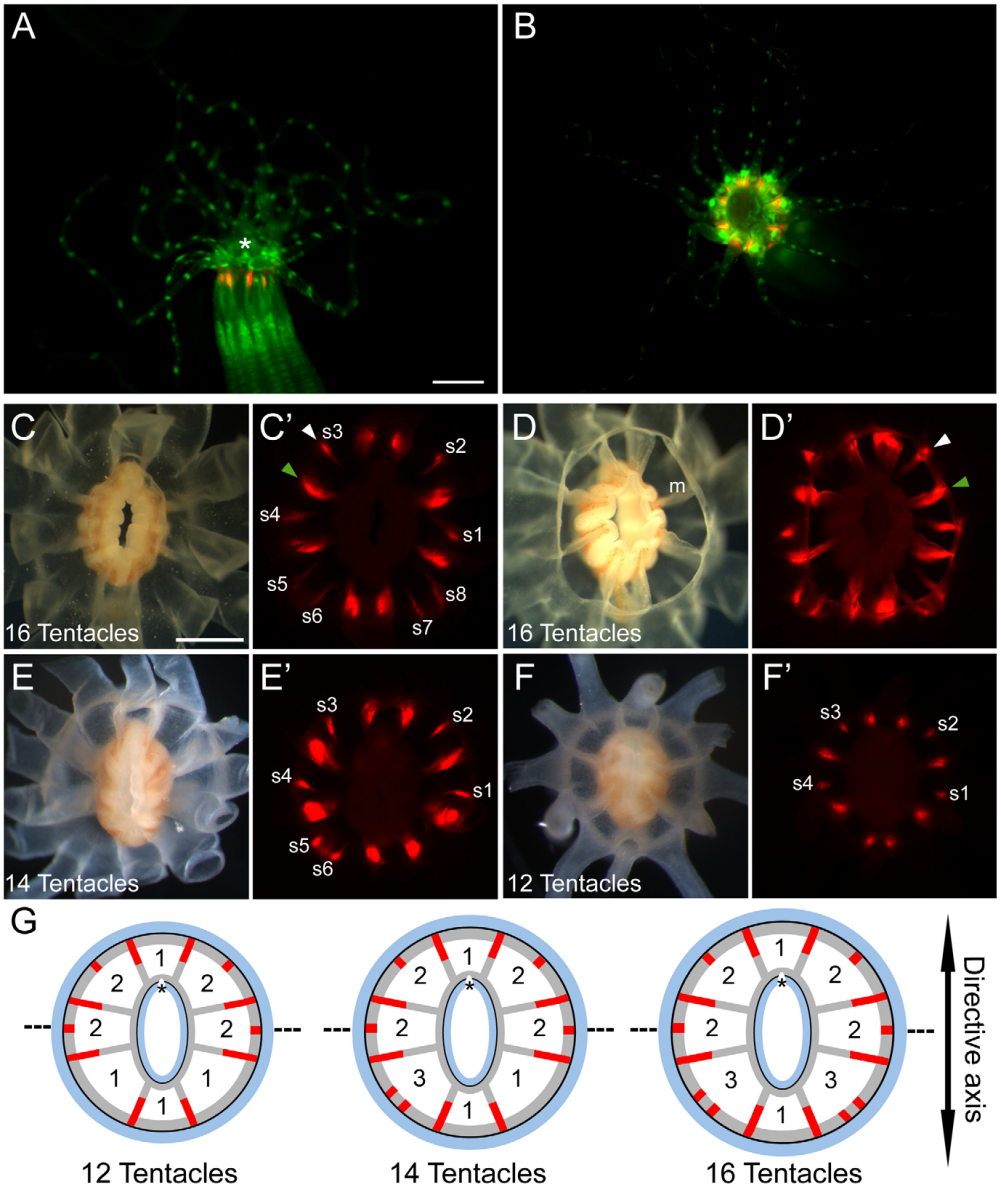
Description of the endogenous fluorescence of *Nematostella*. (A) Lateral and (B) oral views of a *Nematostella* adult under fluorescent illumination using GFP (*green*) and Texas Red (*red*) filters. The oral pole is indicated by an asterisk in A. (C–F) Decapitated oral poles of polyps viewed under white light and fluorescent illumination. The number of tentacles is indicated in each panel. To facilitate observation, the tentacles were surgically removed. (D) An aboral (inverted) view of the sample in C, clearly showing the eight endodermal mesenteries (*m*). (C′–F′) Show the pattern of the red fluorescence for each decapitated polyp, characterized by both short (*s1–s8*, *white arrowhead*) and long (*green arrowhead*) fluorescent domains. (G) Diagrammatic cross-section through the oral pole, summarizing the pattern of the red fluorescence in polyps with 12-, 14- and 16-tentacles, showing ectoderm (*blue*), endoderm (*grey*), and red fluorescence (*red*). The number of tentacles present in each radial segment is indicated between mesenteries, and an asterisk indicates the position of the siphonoglyph. The dashed line represents a virtual boundary separating the half of the animal associated with the siphonogliph pole and the opposite half. The scale bars are 1 mm and 0.5 mm in A and C, respectively.

During the course of experiments conducted under fluorescent illumination, we noticed that *Nematostella* adults exhibit endogenous expression of both green and red fluorescence (Figure 1A and 1B). Weak green fluorescence was detected along the body column but became more concentrated at the oral pole. Green fluorescence was also observed along the length of the tentacles in evenly spaced rings most evident on the oral surface, consistent with oral-aboral polarity of the tentacular ectoderm (Figure 1B).

In contrast to the more broadly distributed green signal, red fluorescence was restricted to the oral pole and was not observed in the tentacles (Figure 1A). At the 16-tentacle stage, discrete domains exhibiting high levels of red fluorescence were distributed around the circumference of the mouth and in-between tentacle pairs (Figure 1C and 1D). These domains were of two types: either short or long (Figure 1C′ and 1D′, *white and green arrowheads*, respectively). There were a total of eight long fluorescent domains, each associated with the eight endodermal mesenteries that attach the pharynx to the body wall (Figure 1D and 1D′). While these eight mesenteries are present from early polyp stages, they did not become visibly fluorescent until polyps possessed twelve completely developed tentacles (Figure 1E–F′). The short fluorescent domains were also first detected at the 12-tentacle stage, and also exhibited endodermal localization. However, unlike the long domains, the number of short domains increased with the number of tentacles. We observed four, six and eight short fluorescent domains in polyps with twelve, fourteen and sixteen tentacles, respectively (labelled s1 to s8 in Figure 1C–F′). This stereotyped red fluorescent pattern was consistently observed in all animals analyzed (n = 39). Thus, temporally, there are two patterns of endogenous red fluorescence during *Nematostella* development: **1**) Long endodermal domains associated with the eight mesenteries that appear in unison at the 12-tentacle stage; and **2**) Short endodermal domains situated between tentacles that arise progressively between the 12- and 16-tentacle stage.

*Nematostella* bears two main body axes: the oral-aboral and directive axes. The directive axis is proposed to be analogous to the dorsal-ventral axis of bilaterian organisms, running perpendicular to the oral-aboral axis [19]. Intriguingly, we found that the short red fluorescent domains were asymmetrically distributed along the directive axis (Figure 1G). Within the half of the animal associated with the siphonoglyph pole, there was one short red fluorescent domain in each radial segment bearing two tentacles. In the opposite half, there were two short red fluorescent domains in each radial segment containing three tentacles. These additional domains developed in concert with the formation of additional tentacles. This asymmetric distribution of red fluorescence along the directive axis thereby provides an easy molecular readout of axial polarity of the oral pole. In this sense, simple examination of the pattern of red fluorescence can serve as a marker of the directive axis. Further, polyps with twelve and sixteen tentacles exhibited bilateral symmetry of the red fluorescent pattern, indicating that it is responsive to the positional information dictated by the body axes as well as the temporal process underlying tentacle formation.

Importantly, while red fluorescence was detected at late developmental stages, green signal was observed at earlier stages. Low levels of green signal were observed in both embryos and planula larvae. The green fluorescence became more clearly visible at the four tentacles stage, primarily in the pharynx region (Figure S1B and S1B′). In addition, we sporadically observed 4-tentacle stage polyps displaying low levels of ubiquitous green and red fluorescence (Figure S1). This sporadic fluorescence might be triggered by unknown environmental factors or a consequence of genetic variation within the population.

### Identification of NvFP-7R, a *Nematostella* Red Fluorescent Protein

Cnidarians are well known for the expression of fluorescent proteins, several of which have proven invaluable as vital protein tags in biomedical research [20], [21], [22]. However, no fluorescent proteins have been described in *Nematostella*, and although unlikely, the patterns described above could arise from an exogenous source. We therefore used computational approaches to search for GFP-like proteins encoded by the *Nematostella* genome (NvFPs). Initially, we identified six GFP-like genes that were predicted by Joint Genome Institute (JGI) gene models. We manually confirmed these predictions using TBLASTN searches and identified one additional GFP-like gene (see Materials and Methods), increasing the number of putative NvFPs encoded in the genome to seven (NvFPs1–7; Table 1). By aligning all putative NvFPs with known GFP-like molecules, we found that only four (NvFP1, NvFP2, NvFP4 and NvFP7) contained the xYG chromophore motif typical of GFP family members [23], whereas the rest lacked a recognizable tripeptide chromophore sequence (Figure S2). In addition, protein structure homology modelling suggested that only NvFP1 and NvFP7 are likely to adopt an 11-strand β-barrel structure similar to that of GFP [24], [25]. Lastly, RT-PCR analysis using total RNA extracted from adult polyps showed that NvFP7 was expressed at detectable levels while NvFP1 expression was not (Figure S3). Consistent with these results, EST sequences from JGI indicate that the only putative *nvfp* gene with detectable expression is *nvfp7*.

**Table 1:** Predicted GFP-like proteins encoded by the *Nematostella* genome.

Gene	JGI Gene Model	Scaffold	Location: Start-End	Number of exons	11-strand β-barrel structure	Tripeptide Chromophore sequence	EST
**NvFP1**	fgenesh1_pg.scaffold_58000086	58	782443–788852	5	Yes	KYG	No
**NvFP2**	e_gw.58.10.1	58	777397–774846	4	No	FYG	No
**NvFP3**	gw.12.10.1	12	976929–978470	3	No	SYG	No
**NvFP4**	-	12	967160–972956	5	No	-	No
**NvFP5**	e_gw.3.8.1	3	2544673–2546776	3	No	-	No
**NvFP6**	gw.2423.4.1	2423	1641–2569	2	No	-	No
**NvFP7**	fgenesh1_pg.scaffold_68000063	68	750162–755799	5	Yes	SYG	Yes

Using the gene finder FGENESH, we predicted that NvFP2 has at least 4 exons instead of 2 exons predicted by JGI. By RT-PCR, we were able to amplify only a part for the first predicted exon of NvFP7 (see Materials and Methods).

**Figure 2:**
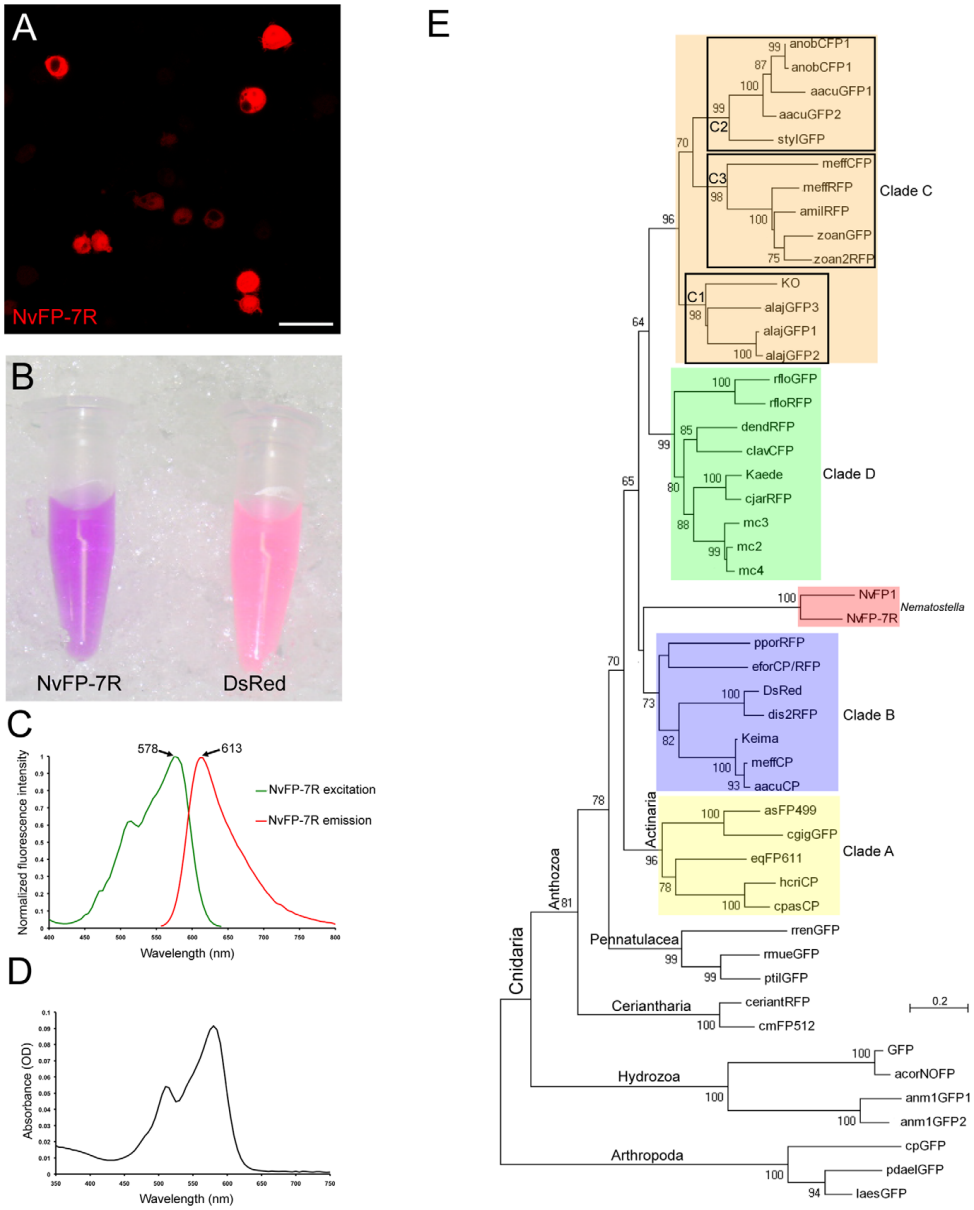
Identification of a novel red fluorescent protein (NvFP-7R) in *Nematostella*. (A) *Drosophila* S2 cells expressing NvFP-7R (scale bar = 25µm). (B) Purified protein solutions of NvFP-7R and DsRed. (C) Excitation and emission spectra of NvFP-7R. (D) Absorbance spectrum of NvFP-7R. (E) Phylogenetic tree showing the position of NvFP-7R with respect to the major anthozoan FP clades (colored clades: A, B, C and D) described in a previous report [13], [15]. In our phylogenetic analysis, the chromo-red protein eforCP/RFP from *Echinopora forskaliana* seems to belong to the clade B. This tree was constructed by the neighbor-joining method with JTT matrix. Bootstrap percentages over 60% are shown on interior branches. Three Arthropod FPs are used as outgroup. The GenBank accession numbers for the protein names used in this phylogenetic analysis are described in [15].

In order to functionally test the fluorescent capacity of putative *Nematostella* GFP-like proteins, we used gene model predictions to synthesize the corresponding cDNAs. All putative *nvfp* cDNAs (except *nvfp6* which is very similar to *nvfp3*) were cloned into pMT/V5-His B vector and expressed under the control of the metallothionein promoter in *Drosophila* S2 tissue culture cells. Strikingly, S2 cells expressing NvFP7 emitted intense red fluorescence when excited with a 561 nm laser (Figure 2A). None of the other *Nematostella* GFP-like proteins produced fluorescence, even when they were excited with other wavelengths (405, 458, 488, 514 and 633 nm). Thus, we focused our analysis on NvFP7, renamed NvFP-7R, for further characterization.

Protein sequence alignment of NvFP-7R with several fluorescent proteins revealed that the tripeptide chromophore of NvFP-7R was identical to GFP (Figure S2), but it shared only about 38% identity with other anthozoan red fluorescent proteins such us DsRed, eqFP611 and Kaede (Figure S4). Due to the lack of strong support for grouping, the phylogenetic position of NvFP-7R with respect to the four major anthozoan FP clades (A, B, C and D) previously described in [13], [15] remained unsolved (Figure 2E). To measure the fluorescence and absorbance spectra of NvFP-7R, we expressed and purified a N-terminal His-tagged form of the predicted protein in *E. coli*. In parallel, we expressed and purified a His-tagged form of the well-characterized protein DsRed as a control. Interestingly, concentrated NvFP-7R protein solution exhibited a visible purple color, similar to mCherry [26] but distinct from the pinkish coloration of DsRed (Figure 2B). NvFP-7R also formed aggregates, suggestive of oligomerization *in vitro*. Spectral analysis using the purified protein revealed that NvFP-7R has a low quantum yield (QY = 0.09) compared to DsRed (QY = 0.63) and other Red fluorescent proteins [15], [27]. At the same time, purified NvFP-7R-His exhibited interesting excitation/emission characteristics measured by spectrophotometery, with an excitation_max_ of 578nm (with a shoulder at 515nm) and an emission_max_ at 613nm (Figure 2C and 2D). These fluorescence qualities were distinct from natural red fluorescent proteins, but reminiscent of some engineered variants. Notably, the excitation_max_ of NvFP-7R was close to mStrawberry (574nm) while the emission_max_ was similar to mCherry (610nm) [26]. Since our spectral measurements were performed with a synthetic form of NvFP-7R predicted from genome sequence and EST data, we next cloned the endogenous gene and repeated the experiments. The endogenous NvFP-7R that we cloned lacked eight amino acids at its N-terminus as compared to the synthetic form (Figure S4), most likely due to cDNA truncation. Nevertheless, its fluorescent proprieties were very similar (if not identical) to the synthetic form (excitation/emission maxima = 575nm/613nm and QY = 0.1).

### NvFP-7R expression, excitation and emission match the endogenous red fluorescence

**Figure 3:**
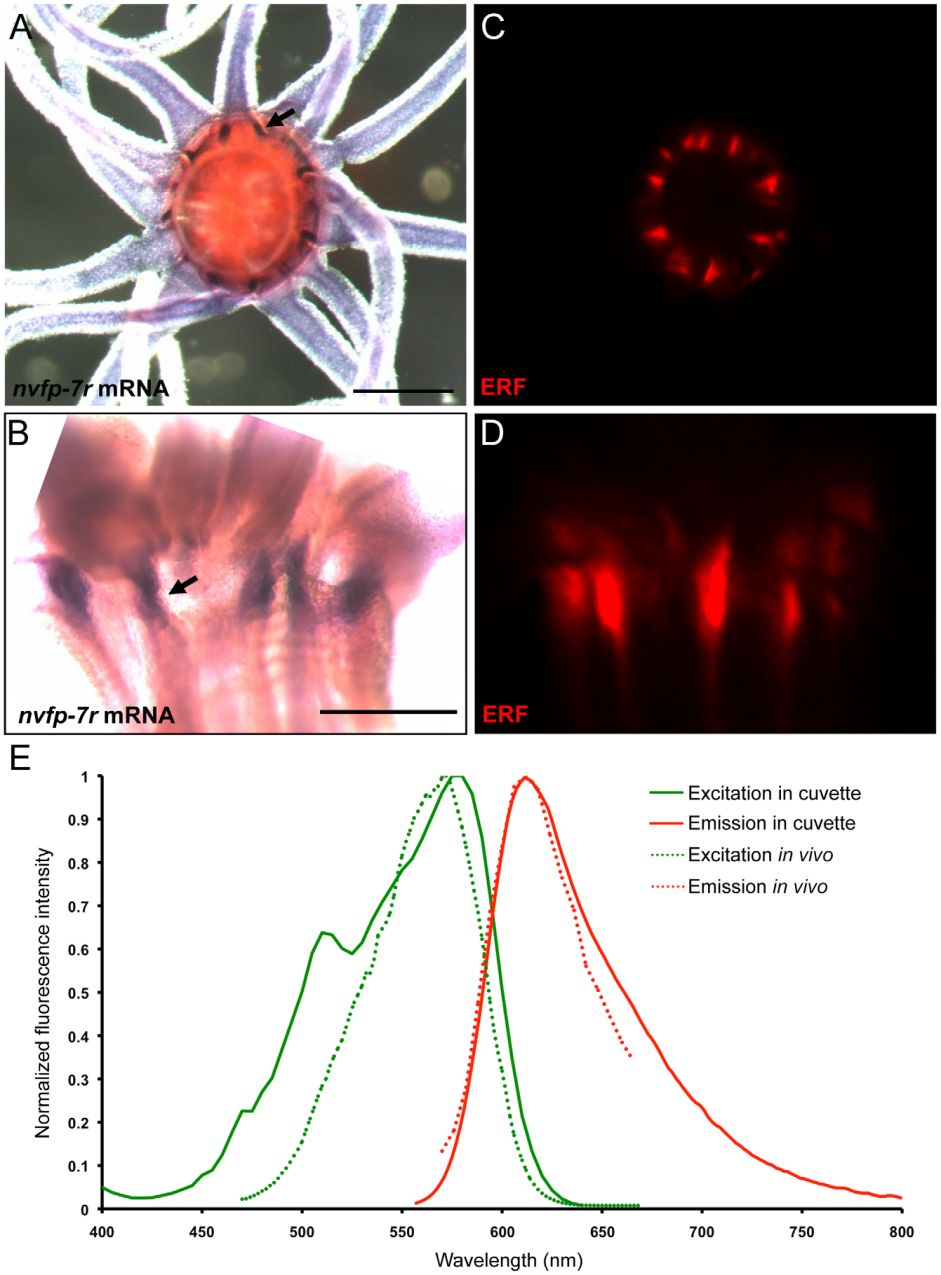
Comparisons between the expression domains and fluorescent spectra of NvFP-7R and the endogenous red fluorescence (ERF). (A and B) mRNA expression of *nvfp-7r* detected by *in situ* hybridization. Black arrows indicate representative domains of NvFP-7R expression. (C and D) The pattern of the endogenous red fluorescence. (A and C) Oral views. (B and D) lateral views. (E) Excitation and emission spectra of NvFP-7Rc and the endogenous red fluorescence. The scale bar is 0.5 mm.

To determine whether the patterned red fluorescence observed at the oral pole of adult *Nematostella* was emitted by NvFP-7R, we generated a probe specific to the *nvfp-7r* mRNA to follow its expression by *in situ* hybridization. Similar to the endogenous red fluorescence, *nvfp-7r* mRNA exhibited endodermal expression localized in discrete domains between the tentacles and also within the eight endodermal mesenteries (Figure 3A–D). This confirms that NvFP-7R is expressed in the exact cell population that emits a red signal under fluorescent stimulation.

As an additional test of whether NvFP-7R is responsible for the endogenous red fluorescence observed *in vivo*, we next used a white laser to excite the *Nematostella* oral pole with a continuous spectrum including all wavelengths from blue to infrared. We thereby determined excitation and emission maxima for the endogenous protein. Focusing on the region where we detected the red fluorescence, we found that the excitation and emission maxima of the endogenous red closely matched NvFP-7R at 572 and 612 nm, respectively. The shape and the peaks of the spectra measured *in vivo* were very similar to those determined for NvFP-7R *in vitro* (Figure 3E). Taken together, our *in situ* and spectral data strongly support the conclusion that the red fluorescence observed at the oral pole of *Nematostella* is emitted by NvFP-7R.

### Unexpected complexity of NvFP-7R expression in the oral endoderm

**Figure 4:**
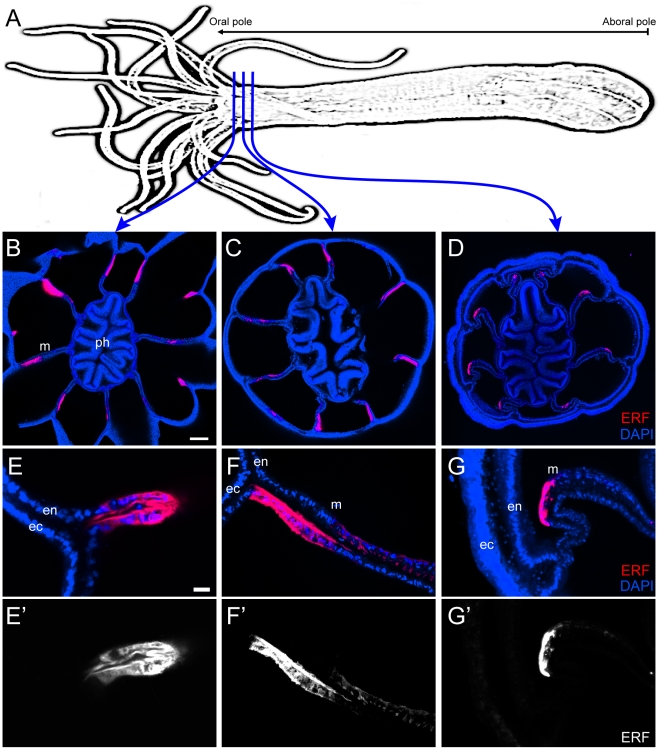
Expression pattern of endogenous red fluorescence in the oral endoderm. (A) Schematic drawing of *Nematostella* showing the position of the cross sections shown in the panels B, C and D. The oral-aboral axis is indicated. (B, C and D) Endogenous red fluorescence observed in increasingly aboral cross-sections through the oral pole. Nuclei are labelled with DAPI (*blue*). (E and E′) Pattern of red fluorescence in the folded region separating tentacles observed at the level of the cross section in the panel B. (F to G′) Pattern of red fluorescence in the mesentery at the level of C and D cross sections. Panels B, C, and D are projections of confocal z-stacks. The scale bars are 75µm and 10µm in B and E, respectively. Abbreviations: *en*, endoderm; *ec*, ectoderm; *m*, mesentery; *ph*, pharynx.

**Figure 5:**
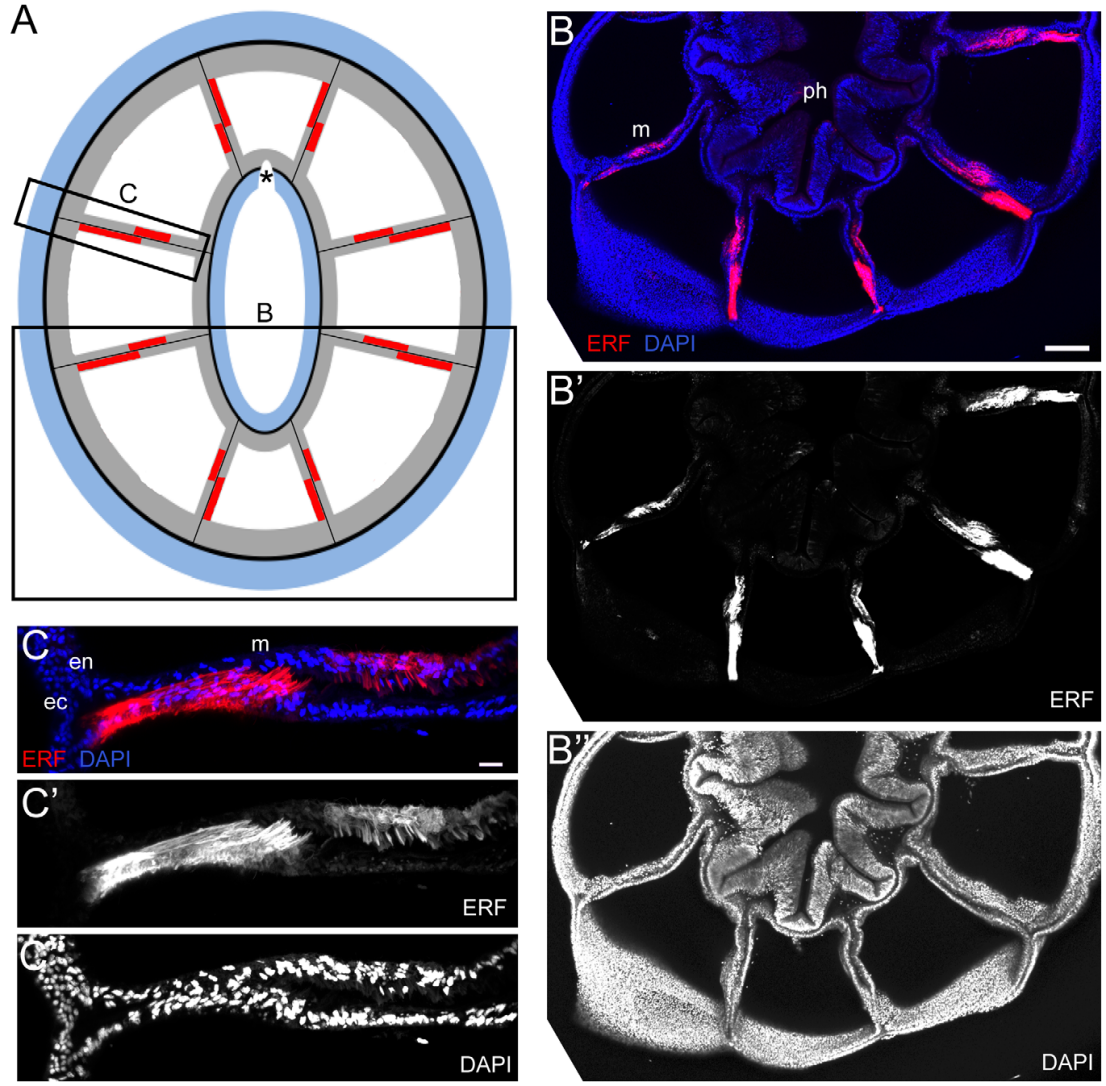
The orientation of the complementary expression domains of NvFP-7R in the two apposed cell layers forming each mesentery. (A) Diagrammatic cross-section through the oral pole showing the expression pattern of NvFP-7R; *B* and *C* indicate the relative position of the confocal images shown in the corresponding panels. (B–B″) Z-projections of a cross section through the oral pole, revealing complementary expression of endogenous fluorescence of NvFP-7R (*red*) in the apposed epithelial comprising each mesentery. (C–C″) Detailed view of the complementary domains in an individual mesentery. Nuclei are labelled with DAPI (*blue*). The scale bars are 100µm and 25µm in B and C, respectively.

**Figure 6:**
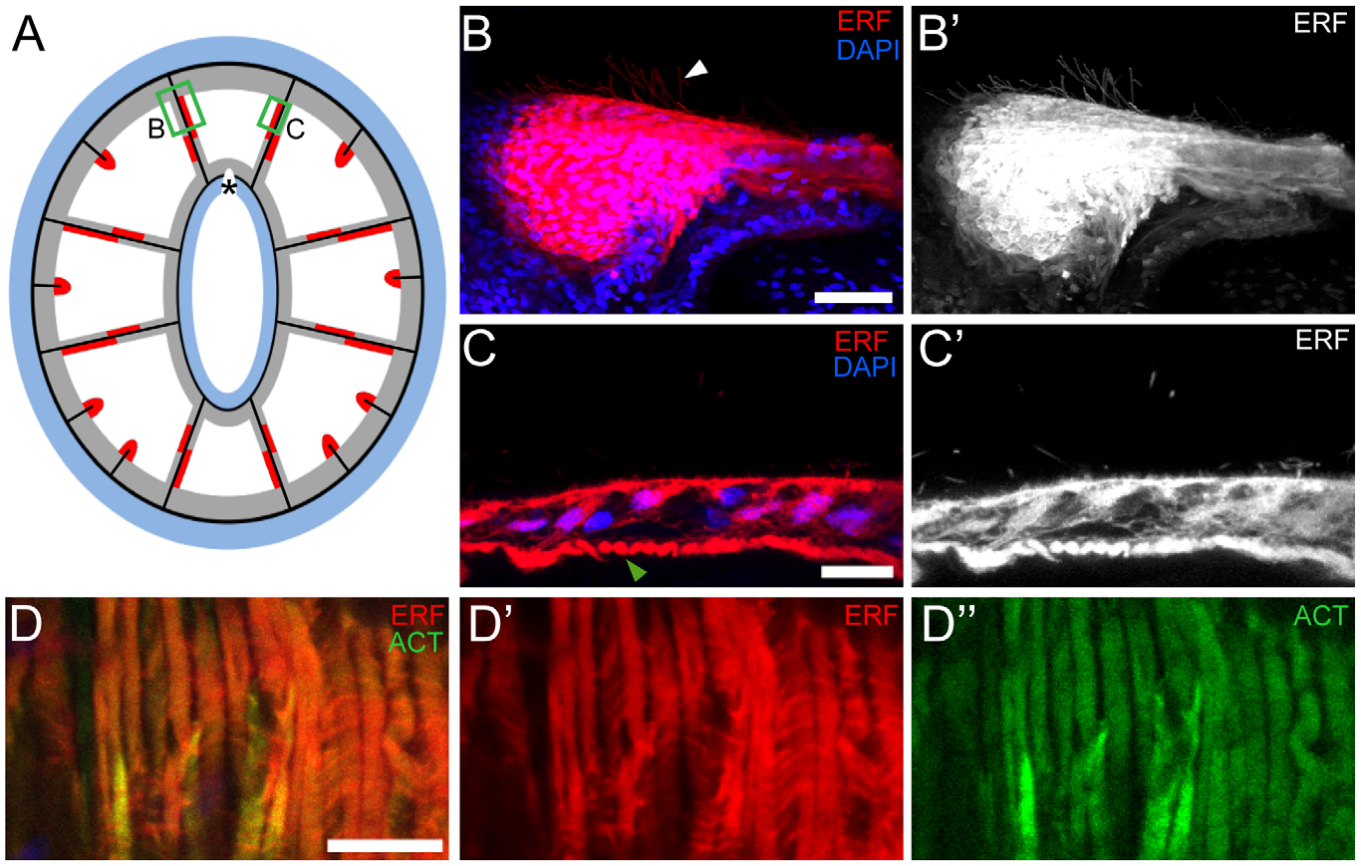
Summary of the expression pattern of the endogenous red fluorescence. (A) Diagrammatic cross-section through the oral pole, showing the expression pattern of NvFP-7R in an adult animal at the 16-tentacle stage; *B* and *C* indicate the relative position of the confocal images shown in the corresponding panels. (B and B′) Z-projections of confocal images of a single mesentery. Fine cellular structures, presumably cilia, project from the apical side of the mesentery (*white arrowhead*). (C and C′) Cross-section through a single cell layer of the mesentery, showing intense NvFP-7R localization in contractile elements at the base of myo-epithelial cells (*green arrowhead*). (D–D″) Lateral view of the contractile elements at the base of myo-epithelial cells showing co-localization of the endogenous red fluorescence (*red*) and F-Actin (ACT, *green*). Scale bars are 25µm and 10µm in B and (C and D), respectively.

We next took advantage of the red fluorescence emitted by NvFP-7R to detail its expression at the cellular level. Serial cross sections through the *Nematostella* oral region were examined by confocal microscopy (Figure 4A). This analysis confirmed that NvFP-7R was exclusively expressed in the endodermal layer. We also observed that NvFP-7R was uniformly expressed in the folded region separating tentacles, earlier described as short fluorescent domains (Figure 4B and 4E–E′). Surprisingly, in the long fluorescent domains, NvFP-7R was detected in complementary expression domains in the two apposed endodermal cell layers comprising each mesentery (Figure 4C and 4F–F′). Specifically, one cell layer generally had stronger fluorescence than its sister when the cross section was near the tentacles (Figure 4F and 4F′). The orientation of these complementary expression domains in each mesentery is summarised in Figure 5A–C and reveals that the bilateral symmetry of the red fluorescent pattern is also maintained at the tissue level. However, in cross sections further from the oral pole, only one of the two endodermal layers emitted red fluorescence (Figure 4D and 4G–G′). This pattern was consistently observed in the eight mesenteries of all analysed animals. Figure 6A shows a diagram of a cross-section through the oral pole, summarizing the expression pattern of NvFP-7R in an adult animal at the 16-tentacle stage.

Unexpectedly, while analysing the *in vivo* expression of NvFP-7R, its fluorescence illuminated previously unrecognized fine cellular structures protruding from the apical side of each mesentery (Figure 6B and 6B′). These structures could correspond to cilia previously observed in the gastric cavity of *hydra magnipapillata* [28], but it is noteworthy that NvFP-7R is broadly distributed within expressing cells. Further, close observation also revealed a significant accumulation of NvFP-7R in the contractile elements at the base of myo-epithelial cells of the mesentery (Figure 6C–D″). To our knowledge, this is the first description of the subcellular localization of an endogenous fluorescent protein and we can only speculate as to the functional implications. Nevertheless, our observations are possibly consistent with a spatially compartmentalized function for the protein *in vivo*.

## Discussion

In this article, we described the spatiotemporal expression dynamics of a novel red fluorescent protein during *Nematostella* development. Temporally, we found that the *Nematostella* life cycle comprises two fluorescent phases: 1) a mono-fluorescent phase characterized by the presence of green fluorescence during early polyp stages; and 2) a bi-fluorescent phase starting at the 12-tentacle stage, defined by the appearance of red fluorescence in the oral endoderm. While the biological functions of the green and red fluorescence are unknown during these stages, we note that the transition from mono- to bi-fluorescence coincides with the sexual maturation of the polyps [29]. Thus, the emergence of the red fluorescence might be a phenotypic trait of mature adults.

Spatially, we found that the green fluorescence was concentrated at the oral pole and in the tentacular ectoderm, while the red fluorescence was strictly localized in the oral endoderm. Thus, it seems that there is a bias in the localization of the green and red fluorescence at the oral pole. In the wild, *Nematostella* is a burrowing anemone, and only the mouth and tentacles are generally exposed to the environment [6]. Hence, orally polarized fluorescence might reflect an oral-specific function in feeding, camouflage [30], or protection from UV light [31], [32]. However, under laboratory conditions, we occasionally found 4-tentacle stage polyps (5/∼300) transiently expressing ubiquitous green and red fluorescence. Although we do not know what triggers this fluorescence, the phenotype might be a manifestation of a stress response. Injured or compromised coral tissues, for example, have been shown to upregulate fluorescent proteins as an inflammation-like response [33], perhaps because fluorescent proteins exhibit anti-oxidant activity [34]. Alternatively, this sporadic fluorescence could be a result of genetic variation or could reflect an exogenous biological source affecting *Nematostella*.

In order to determine the source of the green and red fluorescence, we searched for homologues of GFP-like genes encoded in the *Nematostella* genome. We identified seven *Nematostella* GFP-like genes. In our assays, only NvFP-7R was functionally fluorescent, emitting a bright red signal. According to structural analysis, five of the predicted NvFPs (NvFP2–6) are not likely to be functionally fluorescent because they do not adopt a β-barrel structure and some of them lack a recognizable tripeptide chromophore sequence characteristic to the GFP family. Surprisingly, NvFP1 did not fluoresce despite the fact that it exhibited hallmark structural features of the GFP family. One possibility is that these GFP-like genes have lost their fluorescent capacity but are retained in the *Nematostella* genome for other unknown functions. Some of them may simply be in the process of becoming pseudogenes. Alternatively, because of the lack of EST data, we cannot exclude the possibility that our gene models were either incorrect or incomplete. Nevertheless, even if we mis-annotated another red protein encoded in the *Nematostella* genome, this protein should have the same spectral characteristic as NvFP-7R because our spectral analysis of the endogenous red fluorescence showed a single peak for both excitation and emission spectra.

While we identified a novel red fluorescent protein (NvFP-7R) and its corresponding locus in the *Nematostella* genome, we could not identify the source of the green fluorescence. Two mutually exclusive hypotheses may explain this: 1) The green fluorescence could be emitted by a small molecule or a non-GFP-like protein; or 2) Due to difficulties in the assembly or annotation of the *Nematostella* genome, we could have missed or mis-annotated a putative green fluorescent protein.

To confirm that the red fluorescence observed at the oral pole of *Nematostella* is emitted by NvFP-7R, we showed that both the mRNA expression pattern and spectral properties of NvFP-7R were very similar to the endogenous red fluorescence. Intriguingly, we also observed that expression of the *nvfp-7r* locus follows the positional information instructed by *Nematostella* body axes. This is based on the fact that the red fluorescent pattern of NvFP-7R displayed both bilateral symmetry and asymmetric expression along the directive axis. It has previously been shown that *Nematostella* bears some cryptic genetic and morphological signs of bilaterality and dorso-ventral polarity, particularly at earlier developmental stages [3], [19]. Here, in late polyps and mature adults, we have identified an endogenous molecular marker that clearly illuminates the axial polarity of the oral pole of *Nematostella* during later developmental stages. Another level of complexity in NvFP-7R expression was observed in the two apposed cell layers forming each mesentery. Although these cell layers have no obvious morphological differences at the oral pole, the complementary expression of NvFP-7R reveals an unexpected complexity of gene expression regulation in these structures. This represents another interesting example of spatial complexity associated with *Nematostella* tissues that is not apparent at the morphological level. Finally, these findings reveal some important considerations associated with the use of fluorescent molecules for live imaging, primarily during late stages of *Nematostella* development.

## Materials and Methods

### Culture conditions

*Nematostella vectensis* used in this study was obtained from Mark Martindale and Craig Magie (University of Hawaii). This strain was originally collected from the Rhode River in Maryland, USA [7]. *Nematostella* adults were housed in glass dishes in the dark and maintained in non-circulating 12 ppt artificial seawater at 17°C with daily feedings of fresh *Artemia* and 50% water changes. The adults were spawned every three weeks following a previously established protocol [8].

### Bioinformatics

The Joint Genome Institute assembly (JGI) of the *Nematostella* genome (http://genome.jgi-psf.org/Nemve1/Nemve1.home.html), *Nematostella* v1.0 gene models, and JGI EST clusters were searched using GFP, DsRed and eqFP611 as queries utilizing TBLASTN or BLASTP parameters to isolate potential GFP-like genes (Table 1). NvFP4 was identified by a TBLASTN search of the *N. vectensis* genome, using previously annotated *Nematostella* GFP-like proteins. Gene structures were predicted using the FGENESH program [35]. Multiple sequence alignments were performed using the MUSCLE program [36]. The protein structure homology analysis was conducted by using the SWISS-MODEL automated comparative protein-modeling server (http://swissmodel.expasy.org). For phylogenetic analysis, multiple protein sequence alignments were built using ClustalW [37]. Neighbor-joining reconstructions were performed with the MEGA4 program [38] with protein Poisson distances or JTT matrix using 10,000 bootstrap replicates.

### Cloning and expression of the *Nematostella* GFP-like genes

The predicted genes of NvFP1, NvFP2, NvFP3, NvFP4, NvFP5 and NvFP7 were synthesized by GenScript (Piscataway, NJ). The codons were optimized for gene expression in *Drosophila*. The synthesized genes were cloned into pUC57 plasmid EcoRV site. *Nematostella* GFP-like genes were PCR-amplified from synthesized cDNAs with gene-specific primers carrying EcoRI and XhoI sites and cloned into *Drosophila* S2 cells expressing vector (pMT/V5-His B). The *Drosophila* S2 cells were transfected with these vectors using Effectene Transfection Reagent Kit (QIAGEN) following the manufacturer's instructions. The transfected S2 Cells were induced with 1 mM CuSO_4_. Cell imaging was performed 20–48 hours post-induction.

To clone the endogenous NvFP-7R cDNA, total RNA was isolated from the oral pole of polyps displaying red fluorescence using the RNeasy Kit (Qiagen). NvFP-7R was amplified from the isolated RNA in two steps. In the first step, we used the One-Step RT-PCR Kit (QIAGEN) with the following primers: *NvFP-7R-5′*: 5′-ATGCATAGGTATCCCGATAACAATTACACACAAGG-3′ and *NvFP-7R-3′*: 5′-CTTCAGCTTGGGCAGCTGCA-3′. In the second step, NvFP-7R was PCR-amplified from a 1/50 dilution of the previous reaction product with the primers: *NvFP-7R-5′Nested*: 5′-ATTACACACAAGGAGCCCATCAAGA-3′ and *NvFP-7R-3′* primers. The amplification fragment was cloned into the pCR2.1 vector from the TOPO-TA cloning kit (Invitrogen) and verified by sequencing. We were not able to clone the full sequence of the predicted NvFP-7R. The cloned NvFP-7R is missing 22bp from the predicted start codon. Both the synthesized and cloned forms of NvFP-7R were subcloned into pET-19b (Novagen) and expressed in fusion with an N-terminal 6× histidine tag in *E. coli* BL21 cells. The proteins were purified from bacterial culture using Ni-NTA Fast Start Kit (QIAGEN) following the manufacturer's instructions.

### *In situ* hybridization

The fixation of polyps was performed as previously described in [39] with some modifications. Adult polyps were relaxed in 7% MgCl_2_ in 12ppt artificial seawater for 10 min, then fixed in fresh ice-cold 3.7% formaldedyde with 0.2% glutaraldehyde in 12ppt artificial seawater for 10 min at room temperature. The oral poles were cut from the body and sliced along the oral-aboral axis to allow efficient penetration of solutions during post-fixation and *in situ* hybridization. Post-fixation was performed in fresh ice-cold 3.7% formaldehyde in 12ppt artificial seawater for 1 hour at room temperature. All oral poles were rinsed five times in PTw (PBS buffer plus 0.1% Tween-20) and stored in 100% methanol at −20°C.

*In situ* hybridization was performed as previously described in [39] using a 0.7kb digoxigenin-labeled riboprobe of NvFP-7R. Probe concentration was 0.1 ng/µl and hybridization temperature was 65°C for 24 hours.

### Sectioning of the *Nematostella* oral pole

Polyps were fixed in fresh ice-cold 4% paraformaldehyde in 12ppt artificial seawater for 1 hour at room temperature. After fixation, polyps were rinsed five times in PTw (PBS buffer plus 0.1% Tween-20). For dissecting scope imaging, the oral poles were cut with a scalpel near the base of the tentacles and directly observed for endogenous fluorescence. For confocal imaging, the oral poles were first cut with a scalpel and then embedded in 2% agarose for 30 minutes. Subsequently, a Leica VT1000S vibratome was used to produce 300µm sections. The oral sections were mounted in VECTASHIELD (Burlingame, CA) mounting medium with DAPI.

### Phalloidin staining

Oral poles were fixed and sectioned as described above. The sections were then incubated in 10µl Alexa 488-conjugated Phalloidin (Molecular Probes) in 1 ml PTX (PBS buffer plus 0.1% Triton X-100) plus 0.1% BSA for 1 hour at room temperature. After four rinsing steps with PTX, the sections were mounted in a solution of 70% Glycerol - 30% PTw.

### Spectroscopy

Excitation and emission curves of the purified proteins NvFP-7R and DsRed were measured using Horiba FluoroMax-3 spectrofluorometer. Absorbance values were measured with SpectraMax Plus384Spectrophotometer. The quantum yield was determined by plotting the absorbance of each protein solution versus the integrated fluorescence intensity of the emission curve. Quantum yields were calculated by using Sulforhodamine 101 as a reference standard. The difference in refractive indexes of water and ethanol were taken into account.

### Microscopy

Images in Figures 1 and 3 were captured using Leica MZ 16F scope with QCapture Pro v. 5.1 acquisition software. Confocal images were taken with Leica SP5 AOBS confocal microscope system. *in vivo* spectral analyses of the endogenous red fluorescence were determined using the xyλ scan mode of a Leica SP5 confocal system. Excitation and emission spectra were measured by exciting the oral pole with a white laser tuned from 470 to 670 nm (λ steps = 2 nm) and a 561 nm laser, respectively.

## Supporting Information

Figure S1 Sporadic fluorescence of 4-tentacle polyps. (A, A′ and A″) Mixed population of 4-tentacle polyps displaying normal (*yellow arrowhead*) and ectopic (*white arrowhead*) fluorescence. (B, B′ and B″) 4-tentacle polyp showing a normal green fluorescence, mainly localized in the pharynx (*white arrowhead*). (C, C′ and C″) 4-tentacle polyp displaying ectopic green and red fluorescence concentrated near the oral pole. In panel A, the scale bar is 0.5 mm.
(3.68 MB TIF)

Figure S2 Protein sequence alignment of the predicted GFP-like proteins in *Nematostella* and The GFP protein of the jellyfish *Aequorea victoria*. Beta-strands are shown with black lines. NvFP1 and NvFP7 are highlighted in blue because only these are likely to adopt an 11-strand β-barrel structure similar to that of GFP. Chromophore forming residues are highlighted in green. (*) Residues are identical in all sequences. (:) Conserved substitutions have been observed. (.) Semi-conserved substitutions are observed.
(0.95 MB TIF)

Figure S3 RT-PCR analysis of *nvfp1* and *nvfp7* expression using total RNA extracted from adult polyps. (A) The position of the pair of primers (1F/1R, 1F′/1R′ and 7F/7R) is indicated in the coding regions (*boxes*) of each gene. The primers were designed within regions showing low similarity between *nvfp1* and *nvfp7*. All of these primer pairs should amplify about 500bp of the coding sequence of their corresponding genes. The primer sequences are: 1F: CCGTTAAGAAGGGAGGTCCTTTACC;1R:AATCTGCGTCGTTTTCAACTCTTGT;1F′:ATTCACCGTTAAGAAGGGAGGTC;1R′:GCTTTGCCCCACTCATGCAAC;7F:GCTTTGTGTTGTTAAGGGGAAGCAT;7R:AGTGTCACAGTGCTCGTCGTTGTC. (B) Agarose gel showing that *nvfp7* is expressed while the expression of *nvfp1* is not detected, despite the fact that we used two different pairs of primers for *nvfp1*.
(5.53 MB TIF)

Figure S4 Protein sequence alignment of predicted NvFP-7R and the cloned NvFP-7R (NvFP-7Rc) with several known red fluorescent proteins. Identical residues in all sequences are highlighted in black. Similar residues are highlighted in grey. A Serine is substituted by Aspartate in NvFP-7Rc (*blue line*). The red line indicates the position of the tripeptide chromophore.
(2.76 MB TIF)
