# Supplementary figures and images for: Identification and In Vivo Characterization of NvFP-7R, a Developmentally Regulated Red Fluorescent Protein of Nematostella vectensis
Source: PLoS One. 2010 Jul 27;5(7):e11807. doi: 10.1371/journal.pone.0011807 (PMC2910727; doi:10.1371/journal.pone.0011807)

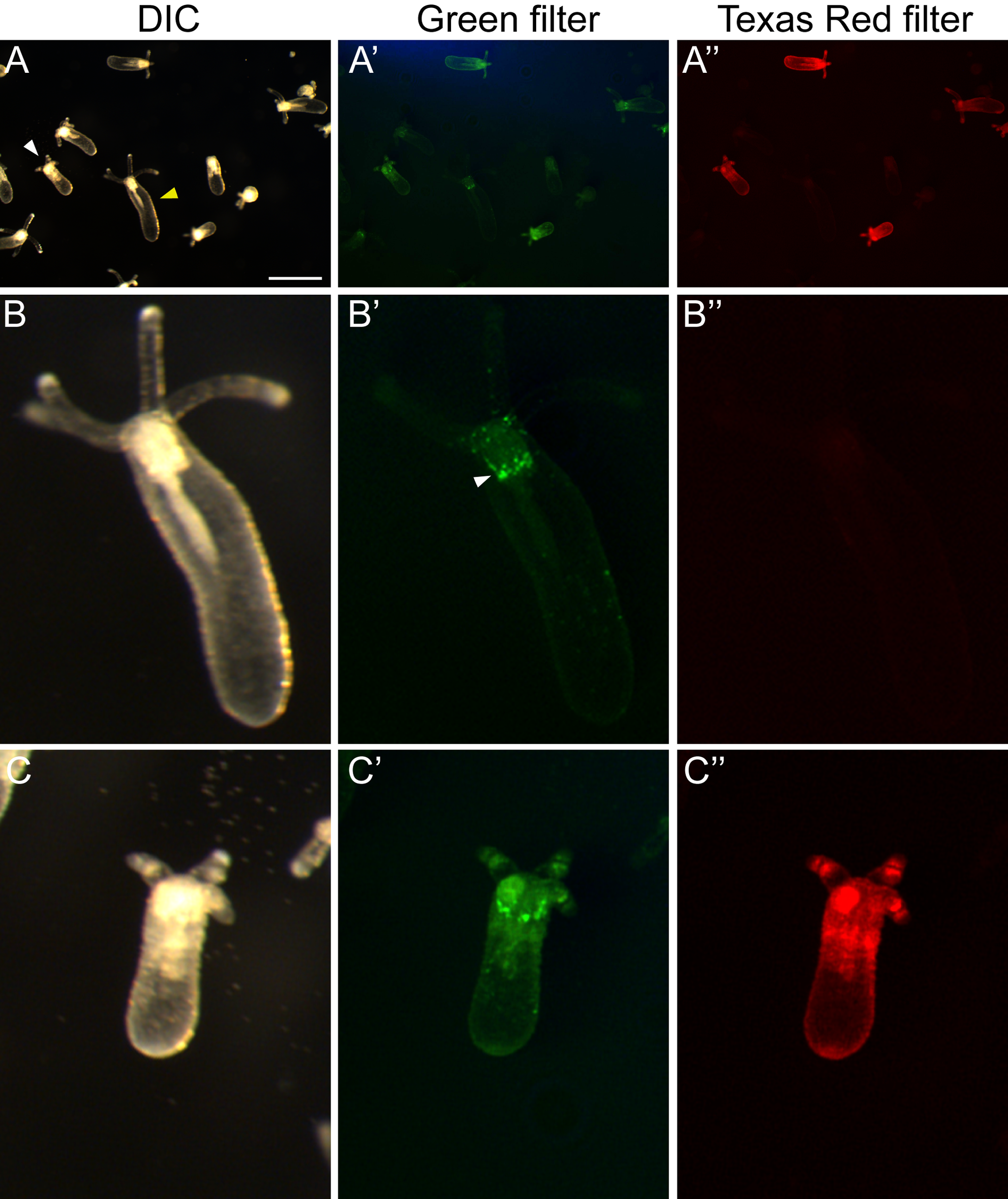

Supplement: Figure S1 — Sporadic fluorescence of 4-tentacle polyps. (A, A′ and A″) Mixed population of 4-tentacle polyps displaying normal (yellow arrowhead) and ectopic (white arrowhead) fluorescence. (B, B′ and B″) 4-tentacle polyp showing a normal green fluorescence, mainly localized in the pharynx (white arrowhead). (C, C′ and C″) 4-tentacle polyp displaying ectopic green and red fluorescence concentrated near the oral pole. In panel A, the scale bar is 0.5 mm. (3.68 MB TIF) [file pone.0011807.s001.tif]

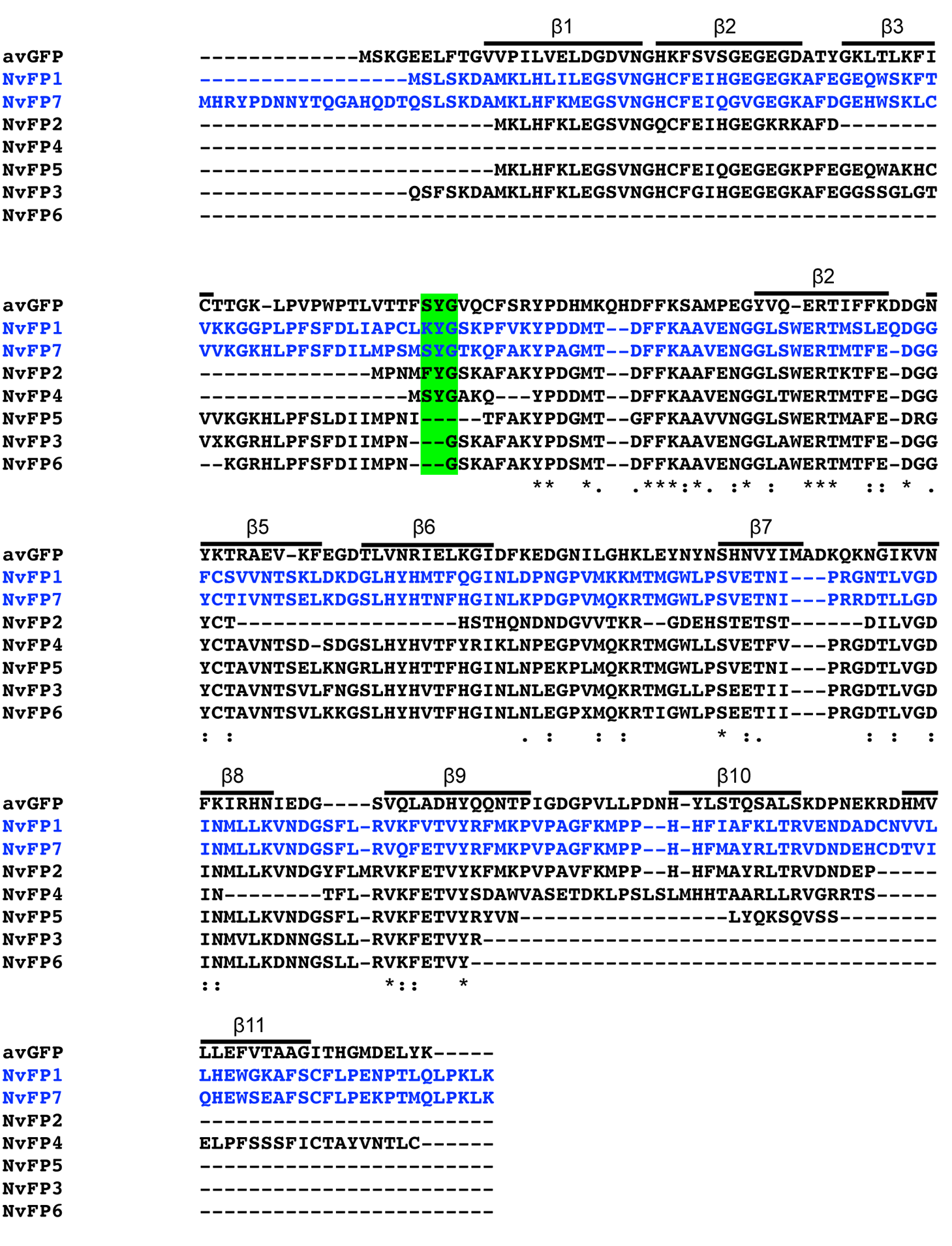

Supplement: Figure S2 — Protein sequence alignment of the predicted GFP-like proteins in Nematostella and The GFP protein of the jellyfish Aequorea victoria. Beta-strands are shown with black lines. NvFP1 and NvFP7 are highlighted in blue because only these are likely to adopt an 11-strand β-barrel structure similar to that of GFP. Chromophore forming residues are highlighted in green. (*) Residues are identical in all sequences. (:) Conserved substitutions have been observed. (.) Semi-conserved substitutions are observed. (0.95 MB TIF) [file pone.0011807.s002.tif]

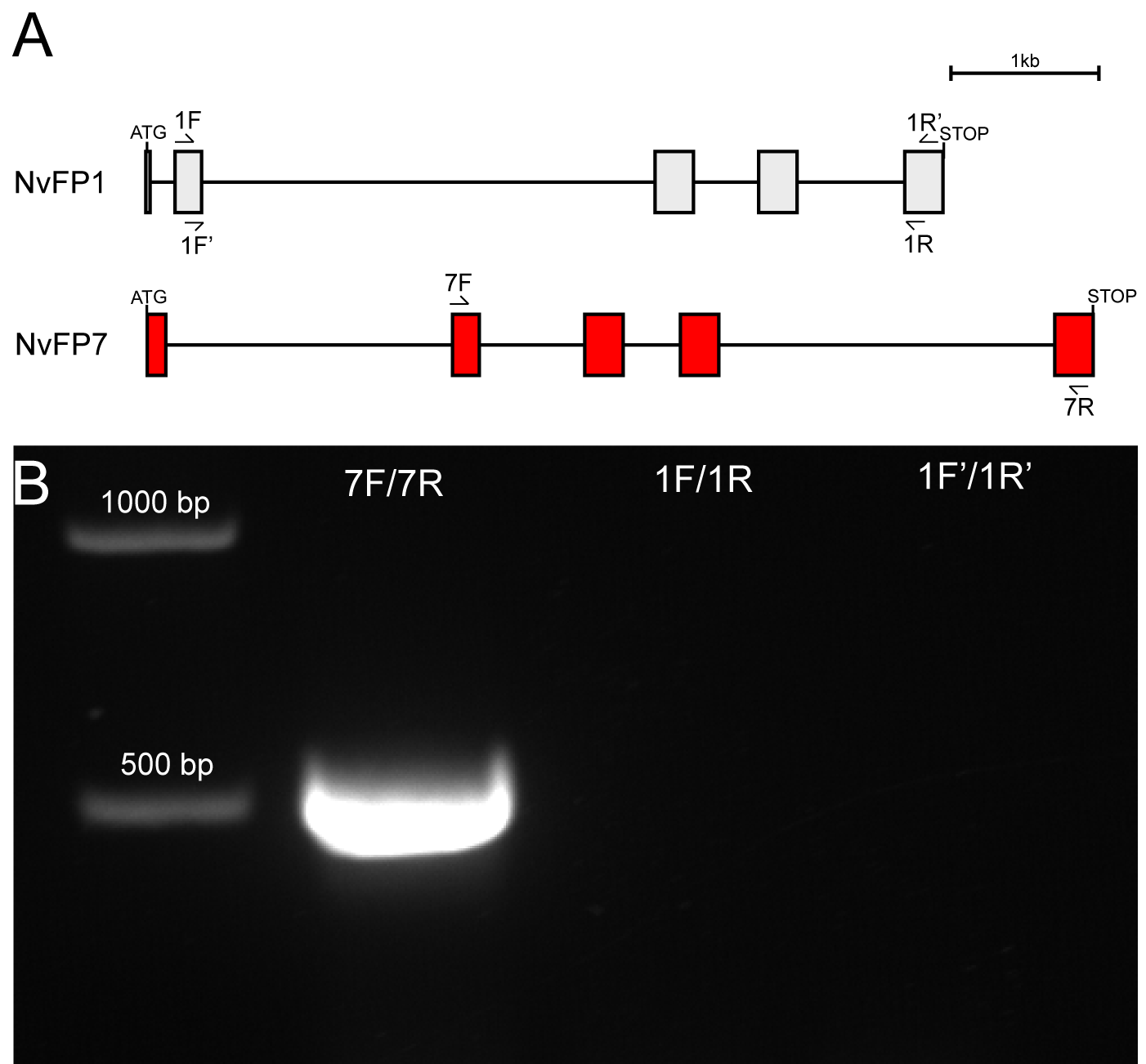

Supplement: Figure S3 — RT-PCR analysis of nvfp1 and nvfp7 expression using total RNA extracted from adult polyps. (A) The position of the pair of primers (1F/1R, 1F′/1R′ and 7F/7R) is indicated in the coding regions (boxes) of each gene. The primers were designed within regions showing low similarity between nvfp1 and nvfp7. All of these primer pairs should amplify about 500bp of the coding sequence of their corresponding genes. The primer sequences are: 1F: CCGTTAAGAAGGGAGGTCCTTTACC;1R:AATCTGCGTCGTTTTCAACTCTTGT;1F′:ATTCACCGTTAAGAAGGGAGGTC;1R′:GCTTTGCCCCACTCATGCAAC;7F:GCTTTGTGTTGTTAAGGGGAAGCAT;7R:AGTGTCACAGTGCTCGTCGTTGTC. (B) Agarose gel showing that nvfp7 is expressed while the expression of nvfp1 is not detected, despite the fact that we used two different pairs of primers for nvfp1. (5.53 MB TIF) [file pone.0011807.s003.tif]

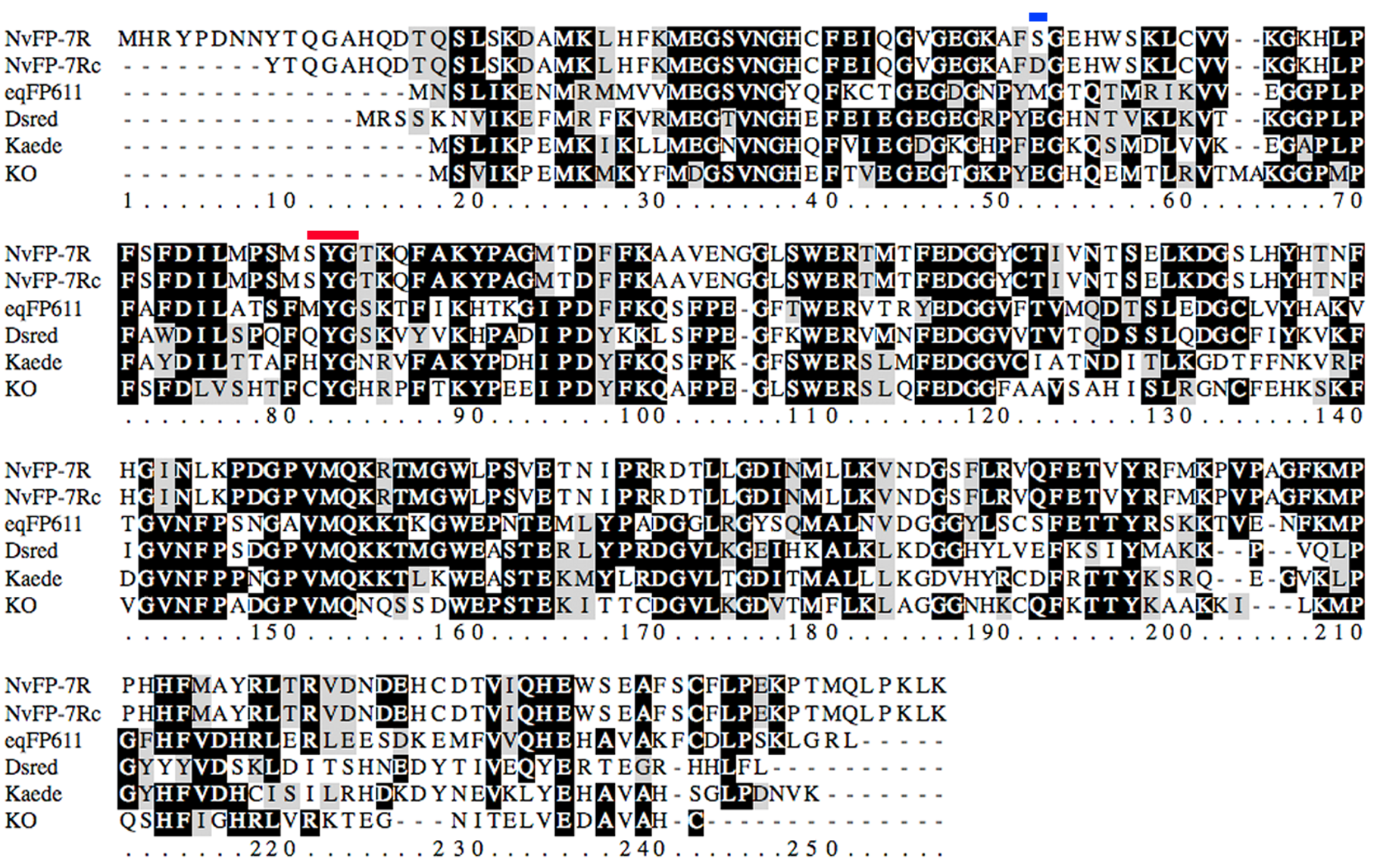

Supplement: Figure S4 — Protein sequence alignment of predicted NvFP-7R and the cloned NvFP-7R (NvFP-7Rc) with several known red fluorescent proteins. Identical residues in all sequences are highlighted in black. Similar residues are highlighted in grey. A Serine is substituted by Aspartate in NvFP-7Rc (blue line). The red line indicates the position of the tripeptide chromophore. (2.76 MB TIF) [file pone.0011807.s004.tif]
